# Happy-Productive worker thesis: The role of work characteristics, gender, and age

**DOI:** 10.1371/journal.pone.0316656

**Published:** 2025-03-06

**Authors:** Jaime A. Bayona, William F. Durán, Jesús Perdomo-Ortiz, Delio I. Castañeda, Carlos A. Valencia, Pauline Fatien Diochon, Diego F. Alvarado

**Affiliations:** 1 Faculty of Economics and Administrative Sciences, Department of Business Administration, Pontificia Universidad Javeriana, Bogotá, Colombia; 2 Grenoble École de Management, Grenoble, France; 3 Hospital Simón Bolívar, Subred Norte, Bogotá, Colombia; St John's University, UNITED STATES OF AMERICA

## Abstract

Happy-productive worker thesis (HPWT) research predicts four configurations depending on well-being and performance levels, one synergistic and three antagonists; however, there has been some discrepancy in the expected results of HPWT, as there are some inclusive results about the specific characteristics that lead to each one of the predicted groups. In this study, we face these discrepancies using a three-configuration model that is more realistic in the organizational context, and exploring how work characteristics, gender, and age can predict workers’ membership in such configurations. We performed multinomial logistic regressions using a sample of 504 Colombian workers and their supervisors from different economic sectors. The results indicated that different work characteristics are associated with the membership of workers in each group, and how this membership varies depending on gender and age group. Our findings offer new research and practice insights about the role of HPWT in HRM (human resources management).

## Introduction

Are happy workers more productive? While the so-called “happy and productive worker thesis” (HPWT) holds that “happy workers” are more productive than their less happy counterparts [1,2], the thesis continues to generate controversy in the face of contradictory research results.

On the one hand, some studies provide evidence of a positive relationship between happiness and performance. In this research, we will use the terms “happiness” and “well-being” interchangeably. When well-being is measured using various constructs and is conceptualized as hedonic well-being—specifically, as job satisfaction—proponents of the model have identified positive associations with motivational aspects such as effort [3], specific measures of managerial performance such as monitoring and follow-up [4], and the quality of performance [5]. On the other hand, some studies find evidence that questions HPWT, as they report weak or spurious relationships between well-being and job performance [6,7].

What could be the causes of this discrepancy? On the one hand, [2] propose, that this lack of consistency in the research results is due to the conceptualization of the relationship as a direct and linear relationship. To address this limitation, they propose understanding this relationship simultaneously by establish one synergistic interaction pattern (i.e., happy - productive) and three antagonistic interaction patterns (i.e., unhappy - unproductive, unhappy - productive, happy - unproductive). However, on the other hand, there is yet another possible alternative that can explain this discrepancy. HPWT traditionally consider the “happy productive worker” as an archetypical person who possess or likes certain characteristics that are common to all workers. This clearly is an excessive generalization, as the needs of each worker are different and not necessary the conditions that lead to high satisfaction and performance are the same for everybody. In particular, gender and age are two demographics that Human Resources Management (HRM) traditionally is interested in, as men and women tend to give priority to different objectives in their work life [8], as well as different age groups are more prone to search specific work characteristics [9].

In light of these disparities between happiness and job performance, as well as new information about synergistic and antagonistic patterns of interaction, this study attempts to provide more support for an alternate HPWT model based on work characteristics [10] and vitamin model [11], which states that some environmental conditions can be prejudicial of beneficial for well-being depending on the actual level of such conditions (e.g., some conditions could deteriorate well-being at low levels, but improve it at high levels).

In particular, with this research, our contributions to the HRM field are twofold: (1) test the differential role of gender and age in an alternative three-category configuration of HPWT, and (2) how the role of work characteristics is presented in each one of those configurations. These contributions will help HR managers better understand the composition of their organization’s workforce and how to improve talent retention depending on their patterns of performance and satisfaction.

### Happy productive worker thesis

Although HPWT and the relationship between positive attitudes and productivity go back from the Hawthorne studies and have been called the “Holy Grail” of industrial psychologists [7,12], these variables’ evidence remains inconclusive. Recently, some researchers have proposed that this is essentially due to three problematic aspects of research on the subject: (a) an excessive emphasis on hedonic measures of workplace well-being at the expense of eudaimonic measures, (b) an inadequate conceptualization of well-being and performance and, (c) a bias towards the investigation of the confirmatory elements of the hypothesis [13].

In particular, [14] propose that productivity and well-being should be sustainable, and their relationship should be synergistic. These ideas emphasize the importance of integrating hedonic and eudaimonic perspectives, revising performance operationalization and conceptualization, and taking a holistic view of the relationship between well-being and performance that considers both the synergistic and antagonistic aspects of the relationship. However, it is also true that the hedonic conception of well-being is the canonical approach inside human resource departments [15].

Peiró [14] propose a model in which the synergistic pattern represents high levels of the two variables. The synergistic pattern with high levels in both variables reveals a virtuous circle of positive reinforcement between them (higher levels of well-being leads to higher levels of performance). On the other hand, antagonistic interaction patterns reveal a tension between the two variables due to higher levels in one compared to the other or reveal possible vicious cycles and negative reinforcement between the variables (e.g., lower levels of well-being lead to lower levels of performance).

There is evidence to support this classification; for example, [16] confirmed the existence of the antagonistic patterns in a sample of young Spanish workers and reported some of their antecedents. Peiró [2] explored the relationships between performance and well-being and synergistic and antagonistic, considering different operationalizations of well-being (i.e., hedonic vs. eudaimonic) and performance (i.e., self-rated vs. supervisors’ ratings). They found four patterns when performance was self-assessed (happy-productive, unhappy-unproductive, happy-unproductive, and unhappy-productive) and three when supervisors assessed it (happy-productive, happy-unproductive, and unhappy unproductive). They also hypothesized that the lack of the unhappy-productive pattern was “due to the fact that employees might be more lenient when self-rating their general performance […and…] it is also possible that, when assessing their own performance, employees’ responses reflect not only their past behavior, but also their expectations of current and future behavior” (p. 16).

It is suggested that for a worker to be included in any of the previous patterns may depend on antecedents related to work, the personal characteristics of the worker, the worker-organization relationship, and antecedents of the organization level [16]. Likewise, it has been proposed that the classification of a worker in the well-being-performance patterns could vary depending on demographic variables [2].

### Work characteristics as antecedents of synergistic and antagonistic patterns

According to job design theory [17,18] job characteristics are essential in predicting job outcomes. Work design models have proposed direct and indirect relationships between work characteristics and work outcomes, both behavioral and attitudinal [19,20]. Contemporary models of job design consider four broad types of work characteristics: (a) task characteristics, (b) knowledge characteristics, (c) social characteristics, and (d) context characteristics [21,22].

Task characteristics refer to how work is done and include autonomy, task variety, task importance, task identity, and job feedback. Knowledge characteristics refer to the skills and knowledge necessary to perform the job, including job complexity, information processing, problem-solving, variety of skills, and specialization. Social characteristics refer to the quality of personal relationships at work and include social support, interdependence, relationships outside the organization, and feedback from other people. Finally, context characteristics encompass those characteristics that refer to the physical and environmental conditions to carry out the work, such as ergonomics, physical demands, the conditions of the work environment, and the use of equipment [21,22].

Research has given consistent evidence of the contemporary model of work design, finding that the characteristics of the work function as antecedents of the results of the attitudinal type of work, such as job satisfaction, as well as the results of the behavioral type of work, such as job performance [20,23]. For example, [19] conclude from their meta-analytic exercise that “more than 34% of the variance in performance and more than 55% of the variance in job satisfaction was a function of the 14 work characteristics investigated herein” (p 1346). In addition, there is evidence of the different patterns of interaction between work characteristics, job performance, and job satisfaction. The first model to report a distinctive pattern of interaction was the job characteristics model [24], which stated that a job with high levels of autonomy, skill variety, task identity, task significance, and feedback from job are *enriched jobs*, a pattern that is related with high job satisfaction and job performance. In addition, the main objective of work redesign was in fact, to assess different profiles of job characteristics and modified them to improve satisfaction and performance.

More recent evidence reporting different patterns of interaction using more work characteristics and more advanced research methods is provided by [25]. Using quadratic equations, they found different configurations for knowledge characteristics (i.e., job complexity, information processing, problem-solving, skill variety, and specialization) on job satisfaction and performance.

In addition to these different patterns, the vitamin model [11] proposed that some environmental conditions at firm levels can be prejudicial to well-being, but at greater levels, they can improve it; these conditions are called “constant effect” conditions: availability of money, physical security, valued social position, supportive supervision, career outlook, and equity. On the other hand, some conditions can deteriorate well-being on small and high levels but can be beneficial at medium levels. These are “additional decrement” conditions: the opportunity for personal control, an opportunity for skill use and acquisition, externally-generated goals, variety, environmental clarity, and contact with others [26].

The work characteristics model and the vitamin model overlap in their contents. Additional decrement characteristics correspond to autonomy, task variety, task identity, job feedback, job complexity, information processing, problem-solving, variety of skills, specialization, interdependence, interaction outside the organization, and feedback from others. On the other hand, constant effect characteristics will be task importance, social support, ergonomics, physical demands, work environment, and use of equipment.

### Role of personal variables on HPWT

Job satisfaction literature shows that, while there is evidence that work characteristics positively affect job satisfaction, evidence indicates that this relationship could be moderated by individual characteristics [9]. As mediators of the association between work qualities and various work outcomes, including features related to well-being, dispositional and demographic variables are critical [27], or societies could reinforce specific interaction patterns as social behaviors in collectivistic cultures [28]. Personality traits like the need for personal growth [29], conscientiousness [30], and neuroticism [31] have been shown to strengthen the effect of job characteristics on satisfaction at work.

**Fig. 1:**
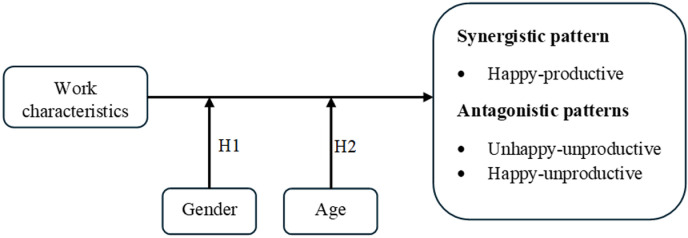
Theoretical model.

According to this evidence, it is possible to support that other personal differences could play an essential role in predicting synergistic and antagonistic patterns (HPWT). Individual characteristics change the way people react to work conditions and modify their attitudinal responses. In this study, we propose that gender and age act as predictors of synergistic and antagonistic patterns of interaction (HPWT). Specifically, it is proposed that patterns of interaction and the work characteristics related to them vary because of workers’ gender and age. A graphical representation of our model is shown in Fig 1.

#### Gender and the HPWT.

Across cultures and job settings, research has shown that work outcomes, like job satisfaction, are related to gender. The so-called gender paradox shows that across cultures and work settings, women are more satisfied with their jobs than their men counterparts [8,32–34] and that adverse work outcomes like stress and burnout also have gender-related patterns [35].

The relation between situational variables (work characteristics), individual differences (e.g., gender), and work outcomes (e.g., job satisfaction and stress) can be explained by the theoretical framework of Clark [32]. He proposes that differences between men and women related to job satisfaction could be explained by departing from the expectation hypothesis. According to this posture, women are consistently more satisfied than men because women have lower career expectations because of gender pay differentials, discrimination, and reduced promotion prospects [8,36], finally [37] found that women are more satisfied than men in restrictive labor markets, supporting the expectations hypothesis.

Clark’s hypothesis did not specify which work conditions are related to women’s higher levels of job satisfaction; [38] found that satisfaction levels vary as a function of specific working conditions and that under similar work conditions to men, women’s job satisfaction levels decrease, finally, [39]showed that women are more satisfied in more flexible settings than their male counterparts suggesting that specific job characteristics affect gender work preferences [8].

From the vitamin model [11], gender is considered a long-term variable that influence well-being and mental health [40,41] as there is evidence that men tend to prefer jobs with higher autonomy and complexity, while women tend to prefer jobs with higher social support and task importance. Following the previous evidence connecting different patterns of interaction between work characteristics, job satisfaction, job performance, and gender, we expected that.

*Hypothesis 1: Synergistic pattern of interaction for women and men (i.e., happy-productive) will be predicted by different work characteristics compared to the antagonistic patterns of interaction (i.e., unhappy-unproductive and happy-unproductive).*

#### Age and the HPWT.

The aging of the workforce is now a predominant phenomenon attracting both researchers and practitioners [42]. Although there is cumulating evidence referring to the relationship between age and different work outcomes, there still is room for further research due to mixed results shown by different investigations [9,43,44]. As people age, interests, and motivation change, they affect human beings’ behaviors and reactions to working conditions and job characteristics. The socioemotional selectivity theory (SES) [45] proposes that age is related to different attitudinal and behavioral changes [9]. According to the concept of future time perspective (FTP), people have different perspectives and expectations about their future and lifespan. Specifically, young individuals tend to see life as an open-ended process, while older ones see this process as bounded and limited. Both perspectives affect how people answer the question about what goals are essential for them [45,46]. According to SES theory, FTP may influence younger workers perspectives to put more attention in job characteristics allowing them to gaining knowledge about the task and professional growth. In contrast, for older workers, FTP may influence their perspectives towards a more social and emotional job characteristics.

In line with SES theory predictions, some work characteristics become more critical because of individuals’ FTP. Younger individuals who believe that there is no time pressure (open-ended perception) will prefer growth-oriented job characteristics. As an example, opportunities for career advancement become significant for younger workers and not for older ones. In contrast, older workers believe that time is not on their side (bonded and limited perception) and focus on more short-time goals and emotional needs [45,47]. Finally, evidence from the vitamin model is scarce [11], but generally, older workers tend to have better job positions with better work conditions. Integrating this rationale with the HPWT model, we expect synergistic and antagonistic patterns to have different work characteristic combinations depending on age.

*Hypothesis 2: Synergistic pattern of interaction for age groups (i.e., happy-productive) will be predicted by different work characteristics compared to the antagonistic patterns of interaction (i.e., unhappy-unproductive, and happy-unproductive)*

Answering these hypotheses will help us identify the role work characteristics of these configurations. To answer the following questions: do workers who fall in the happy-productive, unhappy-unproductive, and happy-unproductive groups tend to have specific work characteristics in their jobs? And are the workers who fall in these groups differ in their gender and age group?

**Fig 2:**
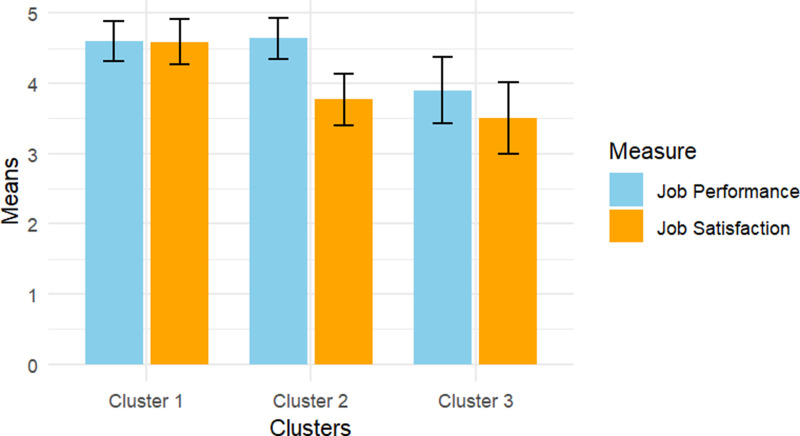
Job satisfaction and job performance by cluster.

## Methods

### Participants

The sample consisted of 532 Colombian workers and their immediate supervisors (*n* =  283), with a worker per supervisor rate of 1.9. Twenty-eight questionnaires were dropped due to incomplete information, leaving 504 useful questionnaires (i.e., response rate =  95%). Table 1 presents the demographic information of the sample.

**Table 1:** Demographic information.

	Workers	Supervisors
Mean age (*SD*)	39.4 (11)	46.9 (10.5)
Gender - Female	58%	45.4%
University degree	50%	80.6%

As shown in Table 2, the sample consists of full-time workers from 20 of the 21 sectoral activities classified in the International Standard Industrial Classification of All Economic Activities [48].

**Table 2:** Distribution of the sample by economic sector.

	N	%
Health Care and Social Assistance	81	16.1
Manufacturing	46	9.1
Professional, Scientific, and Technical Services	46	9.1
Wholesale Trade	39	7.7
Construction	38	7.5
Finance and Insurance	35	6.9
Retail Trade	27	5.4
Accommodation and Food Services	26	5.2
Transportation and Warehousing	23	4.6
Educational Services	19	3.8
Other Services (except Public Administration)	19	3.8
Administrative and Support and Waste Management and Remediation Services	18	3.6
Arts, Entertainment, and Recreation	14	2.8
Mining, Quarrying, and Oil and Gas Extraction	13	2.6
Agriculture, Forestry, Fishing and Hunting	12	2.4
Public Administration	12	2.4
Utilities	11	2.2
Real Estate and Rental and Leasing	11	2.2
Information	6	1.2
Management of Companies and Enterprises	5	1.0
Missing values	3	0.6
Total	504	100

The sample was obtained using a convenience sampling method in organizational behavior and human resource management courses. Junior-level business administration students administered the questionnaires to one or various job incumbents who had worked full time for at least one year in an organization. Job incumbents answer items about work characteristics and job satisfaction; supervisors rated job performance. Each student delivered up to five questionnaires (including both the job incumbent and their supervisor), 83% of students delivered at least one questionnaire. The students received specific training from the authors on how to distribute and soliciting answered questionnaires to job incumbents. This particular sampling strategy is employed when the goal is to sample a wide range of different jobs, as for example [22,49]. Workers and supervisors completed a paper-and-pencil version of the questionnaires, and they were informed about the personal use of all the information provided. Sample was collected from august 4^th^ 2017 to April 26^th^, 2018.

### Ethics statement

The research and ethics committee of the Faculty of Economics and administrative sciences from Pontificia Universidad Javeriana approved the procedure before the study began (original certification in Spanish)

*“That the research project titled “Global Work Design: The role of cultural values on work quality in Colombia,” whose principal investigator is JAB, professor in the Department of Business Administration, has been evaluated and approved by the Research and Ethics Committee of the Faculty of Economic and Administrative Sciences, in its session of February 8, 2017, considering the relevance of the research, methodological rigor, scientific quality, coherence and rationality of the proposed budget, and compliance with scientific, technical, and ethical standards, both national and international, that govern this type of research.*

*The project involves research on human subjects and complies with the Scientific, Technical, and Administrative Standards for Health Research established in Resolution No. 008430 of 1993 and Resolution 2378 of 2008. The risk category to human subjects offered by the proposal belongs to the no-risk research category.*

*The informed consent form prepared for this project includes the aspects required to provide the necessary information to the individuals included in the study, and the principal investigator must ensure the collection of the signed document from each of the study participants.*

*Based on the aforementioned, the Research and Ethics Committee concludes that the project meets all the required quality standards and consequently grants its approval. The respective report is recorded in Act No. 01-2017 of the corresponding session.*

*This certification is issued on February 20, 2017.”*

### Measures

For work characteristics, job performance and well-being we calculated CFA (i.e., Confirmatory factor analysis) using AMOS software (version 26), the likelihood method of estimation and the following fit indices: *χ*^*2*^*/df* ratio, CFI (i.e., Comparative fit index), SRMR (i.e., Standardized root mean square residual), and RMSEA (i.e., Root mean squared error of approximation). For *χ*^*2*^*/df* ratio, a ratio of 2.0 has often been used to indicate good fit. For CFI, higher values indicate better fit, with the value of.90 indicating good fit. In contrast, lower values on both SRMR and RMSEA indicate better fit. With SRMR, a value of.08 indicates good fit, whereas a value of.05 on RMSEA indicates good fit, and values between.05 and.08 indicate adequate fit [50]. We consider a result well-fitting when these cut-off levels are met. Factor loadings and correlations between factors are available from the first author.

### Work characteristics

We measured work characteristics with the Work Design Questionnaire developed by [22] in its Spanish version by [51]. This questionnaire is a self-reporting measure with a 21-factor structure comprising 77 items, which uses a five-point Likert scale ranging from 1 =  *strongly disagree* to 5 =  *strongly agree.* Cronbach’s alpha for the Spanish version was.74 and Cronbach’s alpha for each factor is reported in Table 3. CFA results indicate an acceptable fit for the 18-factor structure (*χ*^*2*^*/df* =  2.05, CFI = .83, RMSEA =  0.05; SRMR = .07). Although the CFI value is low, this can be attributed to the complexity of the model [52]. However, based on the other fit indices (i.e., RMSEA and SRMR) we consider the fit to be adequate. Standardized Regression Weights for the WDQ are presented in S1 Table, while factor correlations are shown in S2 Table. A sample item is “People I work with take a personal interest in me.” We used 18 work characteristics in our study: five task characteristics (autonomy, task variety, task significance, task identity, and feedback from the job), five knowledge characteristics (job complexity, information processing, problem-solving, skill variety, and specialization), four social characteristics (social support, inter-dependence, interaction outside organization, and feedback from others), and four work context characteristics (ergonomics, physical demands, work conditions, and equipment use). Originally, the WDQ considered three sub-dimensions for autonomy and two sub-dimensions for interdependence, however, in the Spanish adaptation of the scale, these variables had some convergent validity issues. Thus, we considered autonomy and interdependence as single variables. We selected the WDQ and its work design structure because it is the most comprehensive model of work design to date [10,19], as it includes different dimensions of work design as the task characteristics of the JCM [24] or the contextual characteristics of the MJDQ [53].

**Table 3:** Descriptive statistics.

Variable	M	SD	1	2	3	4	5	6	7	8	9	10	11	12	13	14	15	16	17	18	19	20
1. Autonomy	3.72	.63	(.89)																			
2. Task variety	4.06	.65	.30^**^	(.79)																		
3. Task significance	4.16	.61	.23^**^	.32^**^	(.68)																	
4. Task identity	3.84	.62	.44^**^	.12^**^	.27^**^	(.75)																
5. Feedback from job	3.84	.64	.42^**^	.26^**^	.29^**^	.48^**^	(.71)															
6. Job complexity	3.07	.74	-.18^**^	.15^**^	.06	-.23^**^	-.13^**^	(.71)														
7. Information processing	3.88	.70	.33^**^	.61^**^	.38^**^	.15^**^	.23^**^	.27^**^	(.73)													
8. Problem solving	3.59	.61	.37^**^	.43^**^	.36^**^	.20^**^	.28^**^	.08	.54^**^	(.53)												
9. Skill variety	3.68	.69	.34^**^	.57^**^	.35^**^	.19^**^	.20^**^	.18^**^	.62^**^	.52^**^	(.81)											
10. Specialization	3.63	.70	.33^**^	.44^**^	.44^**^	.29^**^	.31^**^	.08	.59^**^	.51^**^	.63^**^	(.74)										
11. Social support	3.98	.55	.47^**^	.18^**^	.30^**^	.43^**^	.39^**^	-.12^**^	.27^**^	.32^**^	.22^**^	.25^**^	(.72)									
12. Interdependence	3.33	.63	.08	.32^**^	.23^**^	.13^**^	.22^**^	.10^*^	.38^**^	.32^**^	.28^**^	.31^**^	.10^*^	(.69)								
13. Interaction outside org.	3.68	.89	.17^**^	.18^**^	.29^**^	.08	.13^**^	-.06	.27^**^	.23^**^	.15^**^	.18^**^	.28^**^	.12^**^	(.85)							
14. Feedback from others	3.51	.77	.20^**^	.15^**^	.22^**^	.32^**^	.48^**^	-.11^*^	.13^**^	.20^**^	.11^**^	.21^**^	.37^**^	.17^**^	.07	(.78)						
15. Ergonomics	3.43	.75	.20^**^	-.01	.11^**^	.26^**^	.17^**^	-.07	.08	.02	.04	.03	.28^**^	-.00	-.03	.17^**^	(.42)					
16. Physical demands	2.23	1.02	-.03	.12^**^	-.02	.04	-.01	-.14^**^	-.08	.12^**^	.10^*^	.10^*^	-.04	.09^*^	.08	.02	-.33^**^	(.92)				
17. Work conditions	3.71	.70	.28^**^	.02	.16^**^	.25^**^	.25^**^	-.10^*^	.15^**^	.13^**^	.05	.09^*^	.37^**^	.03	.03	.18^**^	.49^**^	-.28^**^	(.64)			
18. Equipment use	2.95	.76	.09^*^	.32^**^	.16^**^	.16^**^	.16^**^	.02	.33^**^	.34^**^	.40^**^	.49^**^	.06	.30^**^	.09^*^	.15^**^	-.11^*^	.39^**^	-.08	(.59)		
19. Job satisfaction	4.25	.59	.38^**^	.19^**^	.35^**^	.40^**^	.25^**^	-.10^*^	.30^**^	.32^**^	.30^**^	.35^**^	.46^**^	.15^**^	.05	.19^**^	.21^**^	.01	.28^**^	.10^*^	(.84)	
20. Job performance	3.93	.67	.20^**^	.05	.03	.04	.07	.08	.13^**^	.10^*^	.12^**^	.04	.14^**^	.03	-.01	.01	.08	-.07	.12^**^	-.01	.13^**^	(.84)
21. Age	39.38	11.00	.11^*^	.04	.08	.09^*^	.04	-.09	.03	.01	.05	.08	.03	.03	-.02	.12	-.01	.11^*^	-.05	.04	.18^**^	.02

N =  504. Reliability (i.e., Cronbach’s alpha) for each variable is shown in parentheses on the diagonal.

*p < .05. **p < .01

### Job performance

Workers’ job performance was measured using the appraisal of their direct supervisor using a four-item scale developed by [54]. We used supervisor rating because it is the most used rating source in HRM [55]. A Spanish adaptation of this scale was developed for this study using the translation-back-translation technique [56]. CFA results indicate a well-fitting 1-factor structure (*χ*^*2*^*/df* =  2.31, CFI = .98, RMSEA =  0.05; SRMR = .02). The scale uses a five-point Likert scale ranging from 1 =  *strongly disagree* to 5 =  *strongly agree*. Cronbach’s alpha for the Spanish version was.84 (using the sample of this study). A sample item is “The overall level of performance that you have observed for this subordinate is outstanding”

### Job satisfaction

Given that “Organizational scholars theorize that hedonic well-being in the workplace is best represented by job satisfaction because it represents a multi-faceted construct that incorporates both affective and evaluative facets consistent with how general hedonic well-being has been conceptualized and measured” [57, p. 9] we used workers’ job satisfaction as a measure of well-being, employing the 5-item job satisfaction scale reported by [22]. A Spanish adaptation of this scale was developed for this study using the translation-back-translation technique [56]. CFA results indicate a well-fitting 1-factor structure (*χ*^*2*^*/df* =  2.28, CFI = .93, RMSEA =  0.05; SRMR = .02). The scale uses a five-point Likert scale ranging from 1 =  *strongly disagree* to 5 =  *strongly agree*. Cronbach’s alpha for the Spanish version was.84 (using the sample of this study). A sample item is “I like the kind of work I do.”

### Age

Age was measured asking job incumbents about their birth year (*M* =  39.4, *SD* =  10.9). Following [58], we classified our sample in three groups: senior workers, those born before 1968 (N =  97, Mean age =  56.36, SD =  4.51); Middle-aged workers, those born between 1969 and 1980 (N =  136, Mean age =  43.96, SD =  3.59); and Young workers, those born between 1981 and 1999 (N =  258, Mean age =  30.76, SD =  4.61).

### Analyses

In order to obtain the three groups predicted by [2], sample was divided into clusters using the two-step cluster analysis method in IBM SPSS statistics (version 26). The two-step cluster method is based on a two-stage approach that uses log-likelihood as distance measurement method: it undertakes a similar procedure to the *k-*means algorithm in the first stage. In the second step, based on these results, a modified hierarchical agglomerative clustering procedure is carried out that combines the objects sequentially to form homogenous clusters. The two-step clustering algorithm output offers fit information, such as the Bayesian Information Criterion (BIC), and information about the importance of each variable for the construction of a specific cluster. Empirical results indicate that the two-step clustering method shows a near-perfect ability to detect known subgroups and correctly classify individuals into these subgroups [59]. Based on these analyses, the sample was classified, reflecting different job satisfaction and job performance configurations. The selection of the cluster number is based in the change in the BIC value, the ratio of the BIC changes, and the ratio of distance measures (i.e., selecting the number of clusters that obtained largest value in ratio of BIC changes and ratio of distance measures).

To test H1, and H2, we performed a series of multinomial logistic regression analyses. Work characteristics were introduced as predictors of synergistic and antagonistic job satisfaction and job performance groups. This statistical method helps distinguish which of the variables are helpful to predict the group membership and if such predictors were different depending on gender (H1) and age (H2).

## Results

Table 3 shows the means, standard deviation, correlations, and internal consistency values for the measures used in the study. Job satisfaction was significantly related to 16 of 18 work characteristics (mean correlation = .26); there were no significant correlations for interaction outside the organization or physical demands. No signs of multicollinearity were detected, as the highest correction was.63 (between skill variety and specialization). For job performance, 6 out 18 characteristics were related to job performance (mean correlation = .14). A confirmatory factor analysis of the 86 items representing the 20 constructs listed in Table 3 indicated a good fit, as evidenced by *χ*^*2*^*/df* =  2.01, CFI =  0.82, SRMR =  0.08 and RMSEA =  0.05.

In this paper, we control the influence of common method bias on the measures in two ways. This bias can affect the estimation of statistical models because the variables share variance related to the use of the same method. First, we use different sources to measure the independent and dependent variables, thereby decreasing the shared variance by the same method used. Second, we use the common method variance procedure [60]. In the common method variance procedure, we include a general latent factor that represents the shared variance among items in all the constructs. Using this method, we estimated two models: unconstrained and constrained. In the unconstrained model, the items load on their theoretical constructs, while in the constrained model, a latent common factor is included. We compared the change between the unconstrained and constrained models and found no differences between the models in the fit indexes.

### Cluster analyses

The results of the two-step cluster analysis show that the optimal solution consists of three clusters. The auto-clustering algorithm, using the likelihood distance method, indicated that a three-cluster solution was the best model, as it minimized the BIC value (Three-cluster BIC =  201.941; Two-cluster BIC =  258.664; One-cluster BIC =  324.148). The three-cluster solution also had the highest values for the ratio of BIC changes (.866), and the ratio of distance measures (2.908). The average silhouette measure of cohesion and separation was 0.5, indicating fair to good cluster quality. The importance of predictors was 1.00 for job satisfaction and 0.76 for job performance. Using a 15% noise handling, the algorithm discarded 11 cases because of atypical values, leaving 493 cases for further analyses.

The resulting three-cluster configuration was: (1) cluster 1, workers high in job satisfaction (*M* = 4.59, *SD* =  0.32), and high in job performance (*M* =  4.64, *SD* =  0.29), (*N* =  129, 25.6%). (2) cluster 2, workers high in job satisfaction (*M* = 4.60, *SD* =  0.29), and low job performance (*M* =  3.50, *SD* =  0.51), (*N* =  175, 34.7%). (3) cluster 3, workers with low in job satisfaction (*M* = 3.77, *SD* =  0.37), and low job performance (*M* =  3.90, *SD* =  0.47), (*N* =  189, 37,5%). Fig 2 illustrates this distribution by cluster.

Before testing H1 and H2, we used multidimensional logistic regression to explain cluster membership based only on work characteristics. Odds ratios are displayed in table 4. An odds ratio greater than 1 implies that a person in a given category has greater odds of belonging to that cluster analyzed than the comparison cluster; an odds ratio below 1 suggests reduced odds. The multinomial logistic regression analyses identified six work characteristics that explain cluster membership (see Table 4): autonomy, job complexity, interaction outside the organization, task significance, social support, and equipment use. The results show that the model has a good fit (Negelkerke’s *R*^*2*^ = .28, *− 2 log Likelihood ratio [LR]R* =  930.81, *χ*^*2*^ =  139.99, *df* =  36, *p* ≤  0.001).

**Table 4:** Multinomial logistic regression analysis of work characteristics associated with the clusters.

Comparison cluster	1 1		2
Analyzed cluster	2	3	3
1. Autonomy	**1.98** ^*^ **(1.17–3.35)**	1.40 (0.88-2.24)	0.71 (0.41-1.22)
2. Task variety	0.88 (0.51-1.50)	0.95 (0.58-1.56)	1.09 (0.63-1.88)
3. Task significance	1.00 0.61-1.64)	**0.51** ^**^ **(0.32-0.81)**	**0.51** ^**^ **(0.31-0.84)**
4. Task identity	1.01 (0.61-1.67)	0.73 (0.46-1.17)	0.73 (0.43-1.24)
5. Feedback from job	0.89 (0.54-1.47)	1.05 (0.66-1.69)	1.19 (0.70-2.00)
6. Job complexity	**1.46** ^*^ **(1.02-2.09)**	**1.54** ^*^ **(1.07-2.20)**	1.05 (0.71-1.56)
7. Information processing	1.07 (0.60-1.89)	0.69 (0.40-1.20)	0.65 (0.35-1.20)
8. Problem solving	0.99 (0.59-1.67)	0.84 (0.50-1.40)	0.85 (0.48-1.49)
9. Skill variety	1.41 (0.83-2.39)	0.86 (0.52-1.43)	0.61 (0.35-1.08)
10. Specialization	0.60 (0.36-1.01)	0.66 (0.40-1.10)	1.09 (0.63-1.89)
11. Social support	1.27 (0.69-2.35)	**0.35** ^**^ **(0.20-0.62)**	**0.28** ^**^ **(0.14-0.52)**
12. Interdependence	0.98 (0.65-1.48)	0.90 (0.59-1.37)	0.92 (0.58-1.46)
13. Interaction outside organization	0.85 (0.64-1.14)	**1.36** ^*^ **(1.00-1.85)**	**1.60** ^**^ **(1.15-2.22)**
14. Feedback from others	1.04 (0.71-1.50)	1.00 (0.70-1.41)	0.96 (0.65-1.43)
15. Ergonomics	1.25 (0.85-1.85)	1.20 (0.83-1.73)	0.96 (0.63-1.45)
16. Physical demands	1.07 (0.81-1.41)	0.90 (0.68-1.18)	0.84 (0.62-1.14)
17. Work conditions	0.97 (0.64-1.47)	0.77 (0.52-1.15)	0.80 (0.51-1.24)
18. Equipment use	1.04 (0.70-1.55)	**1.71** ^**^ **(1.15-2.56)**	**1.64** ^*^ **(1.06-2.54)**

N =  493. Cluster 1: High job satisfaction and high job performance; cluster 2: high job satisfaction and low job performance; cluster 3: low job satisfaction and low job performance. Odds ratios are shown. 95% confidence intervals are presented in brackets.

*p < .05. **p < .01

The probability of having a synergetic pattern (cluster 1) is more notable when task significance and social support are higher; and when autonomy, job complexity, interaction outside organization, and equipment use are lower in comparison to the two antagonistic groups. This configuration correctly predicted 53.1% of cases of the synergistic group, 33.3% of cases of the group with high job satisfaction and low job performance, and 55.2% of cases of the group with low job satisfaction and low job performance.

### Gender analyses

To test H1, we split the sample into two: one for men and one for women. Odds ratios are displayed in table 5. The multinomial logistic regression analyses identified eight work characteristics that explain cluster membership depending on gender: autonomy, task significance, job complexity, problem-solving, specialization, social support, interaction outside the organization, and equipment use (see Table 5). Results showed a good fit for both women (Negelkerke’s *R*^*2*^ = .34, *− 2 log LR* =  520.99, *χ*^*2*^ =  101.39, *df* =  36, *p* ≤  0.001), and men (Negelkerke’s *R*^*2*^ = .39, *− 2 log LR* =  356.57, *χ*^*2*^ =  86.80, *df* =  36, *p* ≤  0.001). To fully accept H1, the synergistic interaction pattern for men should include different work characteristics than woman; if the patterns are similar in one or more work characteristics we will partially accept H1; and if there are no differences between the groups, we will reject H1a. The same logic applies to H1b (i.e., differences in the synergistic pattern for women with task significance and social support as differentiating characteristics).

**Table 5:** Multinomial logistic regression analysis of work characteristics associated with the clusters by gender.

Comparison cluster	1 1 1 1				2 2	
Analyzed cluster	2	2	3	3	3	3
	Men	Women	Men	Women	Men	Women
1. Autonomy	2.33 (0.86-6.33)	**2.01** ^*^ **(1.01-4.02)**	**2.41** ^*^ **(1.09-5.29)**	1.10 (0.57-2.13)	1.03 (0.37-2.93)	0.55 (0.26-1.14)
2. Task variety	0.62 (0.22-1.78)	0.83 (0.43-1.60)	1.06 (0.47-2.34)	0.76 (0.38-1.50)	1.71 (0.57-5.16)	0.92 (0.45-1.86)
3. Task significance	1.33 (0.53-3.33)	0.71 (0.37-1.35)	0.73 (0.35-1.51)	**0.40** ^**^ **(0.21-0.76)**	0.55 (0.21-1.41)	0.57 (0.29-1.11)
4. Task identity	0.53 (0.22-1.30)	1.25 (0.63-2.46)	0.62 (0.29-1.33)	0.77 (0.40-1.50)	1.17 (0.46-2.93)	0.62 (0.30-1.27)
5. Feedback from job	0.68 (0.29-1.63)	1.08 (0.55-2.09)	0.67 (0.32-1.40)	1.39 (0.70-2.76)	0.98 (0.40-2.43)	1.29 (0.62-2.68)
6. Job complexity	0.84 (0.42-1.68)	**1.96** ^**^ **(1.21-3.16)**	1.40 (0.78-2.52)	1.45 (0.87-2.41)	1.67 (0.79-3.52)	0.74 (0.43-1.27)
7. Inf. processing	1.55 (0.51-4.73)	0.90 (0.44-1.88)	0.85 (0.36-2.00)	0.58 (0.27-1.26)	0.55 (0.17-1.75)	0.64 (0.28-1.46)
8. Problem solving	2.90 (0.91-9.21)	0.72 (0.37-1.38)	**0.30** ^*^ **(0.12-0.77)**	1.63 (0.80-3.32)	**0.10** ^**^ **(0.03-0.37)**	**2.27** ^*^ **(1.09-4.72)**
9. Skill variety	1.32 (0.48-3.66)	1.46 (0.75-2.87)	0.65 (0.29-1.48)	1.05 (0.52-2.14)	0.50 (0.18-1.37)	0.72 (0.34-1.53)
10. Specialization	0.43 (0.16-1.16)	0.78 (0.40-1.52)	1.08 (0.48-2.41)	**0.48** ^*^ **(0.24-0.98)**	2.50 (0.90-7.00)	0.62 (0.30-1.29)
11. Social support	1.25 (0.43-3.66)	1.12 (0.50-2.50)	**0.25** ^**^ **(0.10-0.60)**	**0.36** ^*^ **(0.15-0.83)**	**0.20** ^**^ **(0.06-0.62)**	**0.32** ^*^ **(0.13-0.77)**
12. Interdependence	1.45 (0.68-3.09)	0.90 (0.51-1.57)	0.93 (0.48-1.77)	0.99 (0.54-1.81)	0.64 (0.28-1.47)	1.11 (0.60-2.07)
13. Interaction outside org.	0.67 (0.39-1.16)	0.95 (0.65-1.38)	1.30 (0.79-2.15)	1.39 (0.90-2.16)	**1.94** ^*^ **(1.05-3.61)**	1.47 (0.94-2.31)
14. Feedback from others	1.04 (0.52-2.08)	1.01 (0.64-1.61)	1.51 (0.83-2.72)	0.73 (0.45-1.18)	1.46 (0.69-3.08)	0.72 (0.44-1.19)
15. Ergonomics	1.46 (0.72-2.98)	1.07 (0.64-1.81)	1.52 (0.85-2.71)	0.93 (0.56-1.55)	1.04 (0.48-2.25)	0.87 (0.50-1.50)
16. Physical demands	1.06 (0.67-1.69)	1.09 (0.74-1.60)	0.96 (0.63-1.46)	0.76 (0.50-1.14)	0.90 (0.55-1.49)	0.70 (0.45-1.08)
17. Work conditions	0.91 (0.45-1.86)	1.07 (0.61-1.88)	0.71 (0.38-1.32)	0.73 (0.40-1.31)	0.78 (0.36-1.67)	0.68 (0.36-1.27)
18. Equipment use	1.80 (0.83-3.91)	0.89 (0.53-1.49)	**2.38** ^*^ **(1.17-4.83)**	**1.78** ^*^ **(1.01-3.10)**	1.32 (0.56-3.14)	**2.00** ^*^ **(1.12-3.57)**

N =  284 for women, 209 for men. Cluster 1: High job satisfaction and high job performance; cluster 2: high job satisfaction and low job performance; cluster 3: low job satisfaction and low job performance. Odds ratios are shown. 95% confidence intervals are presented in brackets.

*p < .05. **p < .01

For H1, our results showed that synergistic pattern in the men is associated to higher problem solving, higher social support, lower autonomy, and lower equipment use. In contrast, in the women group the synergistic pattern is related to higher task significance, higher specialization, higher social support, lower autonomy and lower job complexity. These configurations correctly predicted in the men group 61.5% of the cases in the cluster 1, 52.3% in the cluster 2, and finally, 78.2% in the cluster 3. On the other hand, in the women group correctly predicted 53.6% of cases in the cluster 1, 43.5% in the cluster 2, and 78.2% in the cluster 3. According to these findings we partially accept H1.

Moreover, we compare the work characteristics associated with the two antagonistic patterns (cluster 2 and 3). As we expressed before, these clusters differ in the grade of the job satisfaction because it is higher in cluster 2; both clusters have low levels of job performance. Among the job characteristics identified in the men of the cluster 2 are the higher problem solving, and social support, but they have lower interaction outside the organization. In contrast, women of the cluster 2 showed higher social support, and lower levels of problem solving and equipment use.

### Age analyses

To test H2, we split the sample into three: one for senior workers, one for middle-aged workers, and one for younger workers. Odds ratios are displayed in table 6. The multinomial logistic regression analyses identified eight work characteristics that explain cluster membership for age group: autonomy, task significance, feedback from job, job complexity, skill variety, specialization, social support, and equipment use (see Table 6). Results showed a good fit for senior workers (Negelkerke’s *R*^*2*^ = .47, *− 2 log LR* =  158.90, *χ*^*2*^ =  52.63, *df* =  36, *p* ≤  0.05), middle-aged workers (Negelkerke’s *R*^*2*^ = .47, *− 2 log LR* =  224.82, *χ*^*2*^ =  73.81, *df* =  36, *p* ≤  0.001), and younger workers (Negelkerke’s *R*^*2*^ = .35, *− 2 log LR* =  455.54, *χ*^*2*^ =  94.28, *df* =  36, *p* ≤  0.001). If work characteristics associated to the synergistic pattern among senior, middle, and young workers differ we accept H2. Nonetheless, if those groups of workers share work characteristics, we partially accept H2. Finally, if characteristics related in the three groups are the same, we reject H2.

**Table 6:** Multinomial logistic regression analysis of work characteristics associated with the clusters by age groups.

Comparison cluster	1	1	1	1	1	2	2	2	2
Analyzed cluster	2	2	2	3	3	3	3	3	3
Age group	S	M	Y	S	M	Y	S	M	Y
1.Autonomy	1.39 (0.37-5.17)	1.20 (0.38-3.85)	**2.45** ^*^ **(1.09-5.55)**	0.93 (0.26-3.30)	0.81 (0.19-3.37)	**2.28** ^*^ **(1.17-4.42)**	0.67 (0.18-2.45)	0.67 (0.16-2.82)	0.93 (0.41-2.12)
2. Task variety	0.36 (0.09-1.36)	0.79 (0.23-2.67)	1.30 (0.57-2.98)	0.29 (0.08-1.09)	1.93 (0.49-7.66)	1.17 (0.59-2.32)	0.80 (0.21-3.01)	2.46 (0.67-9.04)	0.90 (0.40-2.07)
3. Task significance	3.58 (0.86-14.86)	0.69 (0.23-2.06)	0.90 (0.43-1.86)	0.98 (0.23-4.10)	**0.26** ^*^ **(0.07-0.89)**	**0.43** ^**^ **(0.23-0.81)**	0.27 (0.05-1.36)	0.37 (0.11-1.27)	**0.48** ^*^ **(0.24-0.99)**
4. Task identity	1.01 (0.23-4.49)	0.95 (0.29-3.16)	1.15 (0.54-2.42)	0.44 (0.11-1.79)	0.43 (0.12-1.61)	1.10 (0.58-2.09)	0.43 (0.10-1.94)	0.45 (0.12-1.68)	0.96 (0.44-2.06)
5. Feedback job	**7.06** ^*^ **(1.33-37.53)**	1.18 (0.43-3.22)	0.51 (0.24-1.09)	2.76 (0.51-14.86)	3.11 (0.89-10.85)	0.62 (0.32-1.20)	0.39 (0.08-1.95)	2.63 (0.75-9.29)	1.22 (0.57-2.62)
6. Job complexity	0.68 (0.26-1.73)	2.04 (0.85-4.89)	**2.22** ^**^ **(1.21-4.08)**	0.46 (0.16-1.33)	**5.28** ^*^ **(1.83-15.27)**	**1.73** ^*^ **(1.03-2.92)**	0.68 (0.23-2.06)	**2.59** ^*^ **(1.00-6.68)**	0.78 (0.42-1.44)
7. Information processing	0.76 (0.18-3.09)	0.47 (0.12-1.79)	1.55 (0.63-3.84)	0.91 (0.24-3.43)	0.22 (0.04-1.09)	0.83 (0.37-1.84)	1.20 (0.26-5.63)	0.46 (0.11-1.95)	0.53 (0.20-1.40)
8. Problem solving	1.64 (0.38-7.15)	0.72 (0.23-2.26)	1.11 (0.50-2.47)	3.12 (0.72-13.62)	0.53 (0.13-2.28)	0.72 (0.36-1.44)	1.90 (0.36-9.93)	0.74 (0.18-3.00)	0.64 (0.29-1.44)
9. Skill variety	1.24 (0.21-7.21)	1.10 (0.45-2.69)	1.50 (0.61-3.68)	1.32 (0.23-7.80)	0.77 (0.25-2.35)	0.60 (0.29-1.27)	1.07 (0.16-7.01)	0.70 (0.23-2.09)	**0.40** ^*^ **(0.17-0.98)**
10. Specialization	**0.19** ^*^ **(0.04-0.90)**	1.18 (0.36-3.87)	**0.41** ^*^ **(0.19-0.91)**	0.24 (0.05-1.13)	0.57 (0.14-2.31)	0.75 (0.38-1.50)	1.28 (0.30-5.46)	0.49 (0.11-2.13)	1.82 (0.83-3.99)
11. Social support	0.66 (0.12-3.63)	0.99 (0.27-3.59)	1.87 (0.72-4.86)	**0.18** ^*^ **(0.03-0.96)**	0.44 (0.11-1.79)	**0.34** ^*^ **(0.15-0.78)**	0.27 (0.04-1.77)	0.44 (0.11-1.72)	**0.18** ^**^ **(0.07-0.50)**
12. Interdependence	2.14 (0.69-6.65)	1.09 (0.44-2.67)	0.76 (0.40-1.44)	1.43 (0.42-4.88)	0.44 (0.13-1.44)	1.02 (0.58-1.80)	0.67 (0.18-2.44)	0.40 (0.12-1.34)	1.35 (0.70-2.60)
13. Interaction outside org.	0.61 (0.27-1.35)	1.01 (0.53-1.94)	0.93 (0.60-1.43)	0.95 (0.39-2.32)	1.86 (0.81-4.28)	1.35 (0.89-2.04)	1.56 (0.63-3.89)	1.83 (0.82-4.12)	1.45 (0.91-2.32)
14. Feedback others	0.31 (0.10-1.00)	1.97 (0.81-4.79)	0.81 (0.44-1.49)	0.46 (0.16-1.35)	1.51 (0.62-3.73)	1.17 (0.70-1.95)	1.50 (0.45-4.98)	0.77 (0.31-1.94)	1.43 (0.77-2.67)
15. Ergonomics	1.13 (0.45-2.89)	2.17 (0.92-5.12)	1.21 (0.64-2.30)	1.43 (0.56-3.65)	2.73 (0.95-7.84)	0.89 (0.53-1.51)	1.26 (0.47-3.41)	1.26 (0.43-3.72)	0.74 (0.39-1.40)
16. Physical demands	0.58 (0.26-1.29)	1.08 (0.63-1.82)	1.19 (0.74-1.91)	0.52 (0.21-1.27)	0.73 (0.36-1.47)	1.12 (0.75-1.67)	0.90 (0.35-2.31)	0.68 (0.34-1.36)	0.94 (0.59-1.48)
17. Work conditions	1.07 (0.35-3.26)	0.93 (0.40-2.19)	1.11 (0.56-2.18)	0.58 (0.20-1.72)	0.46 (0.18-1.22)	0.98 (0.55-1.75)	0.54 (0.16-1.81)	0.50 (0.19-1.31)	0.89 (0.45-1.77)
18. Equipment use	0.77 (0.27-2.29)	1.58 (0.67-3.68)	1.22 (0.66-2.25)	1.49 (0.44-5.02)	**5.29** ^**^ **(1.58-17.71)**	**1.74** ^*^ **(1.02-2.97)**	1.94 (0.58-6.49)	**3.35** ^*^ **(0.99-11.26)**	1.42 (0.76-2.68)

S: senior workers, M: middle-aged workers, Y: younger workers. *N* =  97 for Senior workers, 136 for Middle-aged workers, 258 for younger workers. Cluster 1: High job satisfaction and high job performance; cluster 2: high job satisfaction and low job performance; cluster 3: low job satisfaction and low job performance. Odds ratios are shown. 95% confidence intervals are presented in brackets.

*p < .05. **p < .01

Our findings showed that senior workers in the synergetic pattern have higher specialization and social support but lower feedback job. In middle workers we found higher task significance, lower job complexity and lower equipment use. And, finally, young workers in the synergetic pattern are related to higher task significance, higher social support, lower autonomy, lower job complexity and lower equipment use. According to these findings we partially accept H2.

Again, we compare the two antagonistic clusters beyond of the hypothesis proposed. There are not differences between cluster 2 and 3 in the senior workers, middle workers of the cluster 2 have lower complexity and equipment use, and young workers in the cluster 2 have higher task significance, skill variety and social support. This analysis correctly predicted in the senior workers a 50.8% in cluster 1, 43.6% in cluster 2 and 72.6% in cluster 3. In addition, correctly predicted in the middle workers 50.8% in cluster 1, 43.6% in cluster 2 and 72.6% in cluster 3. Finally, correctly predicted in the young workers 55.1% in cluster 1, 31% in cluster 2 and 75.7% in cluster 3.

## Discussion

### Work characteristics and HPWT

This study aimed to examine the effect of gender, and age on the synergistic and antagonistic patterns of the HPWT. We wanted to identify the role of work characteristics of these configurations answer the following questions: do workers that fall in the happy-productive, unhappy-unproductive, and happy-unproductive groups tend to have certain levels of work characteristics in their jobs? And are any differences in gender and age in such groups? In doing so, this work sought to address significant limitations of the HPWT by incorporating contextual and personal characteristics and focusing on the interactions of work characteristics, gender, and age, which allowed us to obtain a far more complex and realistic mapping of how these configurations are formed.

Our results support the modified configuration proposed by [2] with three groups instead of four when job satisfaction is self-rated and a supervisor rates job performance. We consider adequate this three-group configuration more representative of nowadays organizations, as the synergic group is composed of good performers who are satisfied with their jobs and commonly labeled as talented workers. Furthermore, this three-group configuration emphasizes the importance of job satisfaction in workers, as the configuration of unhappy but productive workers did not appear in our sample; this result may lead to further consideration of the relationship between job satisfaction, and job performance, as high performance appears to necessitate some minimum satisfaction threshold to develop a talented workforce fully.

According to our findings, work relevance and social support are significant differentiators from the antagonistic group of low job performance and job happiness. We can say that these two attributes are related to higher levels of job satisfaction, one related to the job itself and the other from the people the incumbent job works with. The first one reflects the “passion” of the work itself, as the higher the passion, the higher the satisfaction and internal motivation to perform better in a task that is important for the worker (task significance). On the other hand, social support can provide friendship, assistance with specific complex tasks, or motivation to continue with daily job responsibilities; additionally, we believe that social support is a highly valued characteristic in the Colombian context, given the country’s collectivist culture. [28] and highly appreciate social interaction. Those happy and productive workers feel and behave in this way because they are highly motivated by their job (a passion that is reflected in the task significance) and by the people they are working with (social support).

But higher values of task significance and social support tell just half of the story. To be happy and productive, the worker needs to have a job not so complex, with low equipment usage and low interaction outside the organization. For job complexity, if it is too little, the worker could be bored, and the job satisfaction could fall, but if the job is too complex, workers could feel that they are not well prepared for the job and feel frustrated about it. If the job needs too complex equipment to handle for equipment use, the worker could be frustrated. Finally, interaction outside the organization, too little of this characteristic could be related to a common understanding of the organizational mission and role in the society. It is good to understand customers, suppliers, and other stakeholders’ points of view that can enrich the work process. However, suppose there is an excessive level of interaction with other people (e.g., customer service). In that case, the emotional costs of these interactions can deteriorate performance and satisfaction and lead to turnover intentions [19].

Finally, concerning the somehow strange pattern of happy and unproductive, autonomy and job complexity are key differentiators. For autonomy, as in classic job design [24], higher levels of autonomy were related to enriched work. However, autonomy must have limits, as if the worker has no boundaries, she could feel “lost” at their job and have no clear feedback about the effectiveness of their work behavior. If these extreme levels of autonomy are paired with a highly complex job, it is expected to have low-performance levels.

Our contribution to the HPWT concerning work characteristics is to have found some key work characteristics that are “typical” of the synergetic configuration: a job that is motivating because it is related to the workers’ passion (i.e., task significance) and offers engaging work and personal relationships (i.e., social support); but that also have adequate levels of autonomy (i.e., low), complexity, equipment use and interaction outside organization that keep the work interesting, motivating and engaging, but that not isolate or frustrate the worker.

### Gender and HPWT

Our findings revealed that work relevance and social support are significant differentiators from the antagonistic group of low job performance and job satisfaction. However, interestingly, it is more present for men than for women, so although in the synergic group, the proportion of women is more significant than in the antagonistic groups, the expectation hypothesis [32] is not enough to explain our results. Given that problem-solving is prevalent in the jobs of happy and productive men, we can speculate that social support can act as a buffer against the adverse effects of increased problem-solving, as social support provides not only friendship but also help and guidance in the workplace. [11].

On the other hand, task significance and specialization are only present for women. This means that happy-productive women have jobs that they feel more passionate about and more demanding jobs in knowledge formation and creation. This dual pattern could indicate that, against what expectation theory states, women are more satisfied because they have more meaningful (task significance) and challenging work (specialization), probably, because of a change in the labor market, where qualified female workers have more presence: “skilled performance is valued not only because it contributes to the solution of problems and progress toward goal achievement but also because it is satisfying in its creative, challenging and/or familiar aspects a job that demand the application of specific knowledge from the worker, give them the opportunity to be more engaged in their work” [61].

For men and women, autonomy and equipment use played the same role; job complexity should be kept low to keep women happy and productive; the effect of interaction outside the organization vanished once the sample was split into men and women, which can be explained as a statistical artifact, as the effect was negative for the general group. However, once we split the sample, the effect disappears.

It is not relevant for women in the case of problem-solving, but it is for men, as happy and productive men tend to have jobs with much more problem-solving than their less happy and productive congeners. The gender differences provide relevant information about slight differences in happy-productive workers that go beyond what we traditionally know about gender differences: men prefer autonomy and skill development, but women tend to prefer social support. However, we should also consider that gender differences are becoming less marked [11], as recent decades have seen structural changes in women’s employment opportunities. Our contribution shows that men tend to have jobs with knowledge characteristics and social support. Women tend to have more significant jobs, have higher social support, and are highly specialized.

### Age and HPWT

Finally, we identified the main work characteristics across three different age groups. For happy-productive older workers, the critical differentiator was social support. This result aligns with the TFP, which suggests that older workers focus more on social and emotional characteristics than on knowledge. However, we also found that compared to happy but unproductive senior workers, the synergistic group exhibits lower levels of feedback from job and tends to report higher levels of specialization. This implies that these workers are generally in positions of higher responsibility, requiring more advanced training, but receive less direct feedback from their job, as is often the case in high-level managerial roles.

For middle-aged workers, the essential characteristics were task significance, job complexity, and equipment use. This leads to a pattern in which happy-productive middle-aged workers have a job in which they are fully committed. They feel passionate about (i.e., task significance). They have low levels of job complexity and equipment use, which we already explained how high complex jobs could need complex equipment.

Finally, for younger workers, the pattern of work characteristics is somehow similar to that of middle-aged workers. Again, they have high task significance (feeling passionate about the job is essential) and low job complexity and equipment levels. However, they also have high levels of social support, which could be understood from the composition of our sample in a Latin American country that highly appreciates social interaction [28], with significantly younger workers. In addition, low autonomy is also essential for this group, which can be related to the initial stage of their careers in which they are in positions with low responsibilities. Finally, in our results, younger workers tend to be more represented in the low job satisfaction and performance (42%), with the lowest proportion in the HPW cluster (22%). This could be related to the initial career stage in which these workers are still in development and probably don’t already haven’t have the competencies to be included in the jobs they aspire to.

In contrast to the SES theory, which states that social support is more important for older workers, social support is equally important for younger and senior workers. The importance of the work is fundamental for middle-aged workers in our Colombian sample. Younger workers, a trend aligned with the talent management movement in which a pivotal aspect of attracting and retaining talent is that workers feel passion for their job.

### Theoretical implications

Our contributions to the HPWT are threefold.

First, we confirm the composition of the synergic and antagonist groups proposed by Peiró et al. (2019), in which only three groups are presented when the supervisor assesses job performance. This modification to the traditional model of HPWT of four groups is more realistic and based on the HRM practices and helps to approach theory and practice.

Our second theoretical contribution to the HPWT is to add a prototypical configuration of work characteristics that are more likely to present in happy and productive workers than in other configurations. This interlink between HPWT and the work characteristics model is based on the pioneering JCM of Hackman and Oldham [29] that associates specific task characteristics to an enriched job correlated with job satisfaction and job performance.

Finally, our third theoretical contribution was identifying differences in the work characteristics configuration depending on gender and age that were not predicted by stablished theories and could be related to cultural differences in the Colombian context and the changing labor market that we have nowadays. Our results show that happy, productive men. However, they have more problem-solving in their jobs and get more social support, which complements the traditional view of men’s preferences for social interaction. It could be true that they prefer autonomy and knowledge characteristics in their jobs. However, at the moment of being included in a group of both happiness and productivity, the role of the social component is critical. This social support role is also detected in the younger group of workers. The theory states that they are more interested in learning new abilities; again, although it could be true, it is also true that young happy-productive workers are more those with higher levels of social support. Again, this highlights the difference between job characteristics preferences and the actual configuration of jobs associated with happiness and productivity.

### Managerial implications

The findings of this study have significant HRM implications, the first of which is the critical role of social support and task significance in having a happy and productive workforce and how this work characteristic can function as enablers of talent retention, as it provides workers with not only personal but also professional support. In addition, the role of task significance is also crucial to maintaining a happy-productive workforce, which is coherent with the talent management literature, especially for strategic workers. This is relevant for selection processes where an emphasis on the congruence between the demands of the job and the candidate’s expectations is essential to detect the perceived significance of the job for the worker.

Although we only assessed certain work characteristics that are more prevalent in this group, and our data is cross-sectional, our results suggest that social support and task significance are key factors for workers to improve their perception of their jobs. We found that the importance of these characteristics is typical among workers with medium levels of both performance and satisfaction. Taking the JCM into account, this suggests that by improving these characteristics (i.e., social support and task significance), managers can focus on work redesign to enhance both performance and satisfaction.

### Limitations and future research suggestions

This study obtained some compelling results, but five limitations restricted it. Being the first to lack more objective performance evaluations, and even though our findings were consistent with those of [2]. Comparing the job characteristics configuration with groups of workers appraised by their direct supervisors or objective assessment methods will be a future avenue of inquiry. This is also related to the lack of an antagonistic group, those with low job satisfaction and high job performance; in our cluster analyses, that configuration was not obtained, but [2] reported it when an objective job performance assessment was obtained. Future research should focus on this missing group and how the work characteristics configuration is presented.

Our second limitation is the use of job satisfaction as the sole measure of well-being. The study uses “job satisfaction” to measure happiness/well-being as noted in footnote 2. While job satisfaction is a valid indicator of well-being within the workplace context, it is a type of domain-specific satisfaction. Further research should consider broader measures of well-being, such as life satisfaction or positive emotions, may provide a more comprehensive understanding of an individual’s overall well-being.

Our third limitation is the sample size, especially for the age comparisons, as for older workers, we could obtain a small sample. This is equally true for newer employees (i.e., those less than 20 years old and who have been incorporated into the workforce), for this reason, results from the age comparisons should be taken with caution. The research focused on this sample will be essential to understand the expectations of this group and if they are different from older workers.

The fourth limitation is the sampling method, where students collected information from workers in organizations. While this approach facilitated data collection, it could limit the analysis of specific worker groups. To mitigate this, we thoroughly reviewed each questionnaire submitted by students. However, for future research, using a sample directly from an organization would allow us to replicate our findings in a more homogeneous work environment.

Finally, this study employs a cross-sectional design, which limits the ability to infer the direction of causality between variables. While the findings indicate associations between work characteristics, gender, age, and well-being/performance configurations, it is not possible to determine whether these factors cause changes in well-being and performance or vice versa. Longitudinal and experimental studies are needed to better establish causal relationships.

## Conclusions

This study employed a three-configuration model of the Happy-Productive Worker Theory (HPWT) to examine how work characteristics differ among worker groups and how gender and age influence these group memberships.

Synergetic pattern (i.e., group of workers with high job satisfaction and high job performance) is more notable when task significance and social support are higher; and when autonomy, job complexity, interaction outside organization, and equipment use are lower.

Synergistic pattern in the men is associated to higher problem solving, higher social support, lower autonomy, and lower equipment use. In contrast, in the women group the synergistic pattern is related to higher task significance, higher specialization, higher social support, lower autonomy and lower job complexity.

Finally, senior workers in the synergetic pattern have higher specialization and social support but lower feedback job. In middle-aged workers we found higher task significance, lower job complexity and lower equipment use. And, finally, young workers in the synergetic pattern are related to higher task significance, higher social support, lower autonomy, lower job complexity and lower equipment use.

## Supporting information

S1 Dataset Study sample.
(CSV)

S1 Table Standardized Regression Weights for the WDQ.
(DOCX)

S2 Table Factor correlations for the WDQ.
(DOCX)

## References

[1] Cropanzano R, Wright TA. When a “happy” worker is really a “productive” worker: A review and further refinement of the happy-productive worker thesis. Consulting Psychology Journal: Practice and Research. 2001;53(3):182–99. doi: 10.1037/1061-4087.53.3.182

[2] Peiró JM, Kozusznik MW, Rodríguez-Molina I, Tordera N. The Happy-Productive Worker Model and Beyond: Patterns of Wellbeing and Performance at Work. Int J Environ Res Public Health. 2019;16(3):479. doi: 10.3390/ijerph16030479 30736334 PMC6388150

[3] Seo M-G, Bartunek JM, Barrett LF. The role of affective experience in work motivation: Test of a conceptual model. J Organ Behav. 2010;31(7):951–68. doi: 10.1002/job.655 21785527 PMC3141585

[4] Hosie P, Willemyns M, Sevastos P. The impact of happiness on managers’ contextual and task performance. Asia Pac J Human Res. 2012;50(3):268–87. doi: 10.1111/j.1744-7941.2012.00029.x

[5] Rothbard N, Wilk S. Erratum: Waking up on the right or wrong side of the bed: Start-of-workday mood, work events, employee affect, and performance. Academy of Management Journal. 2011;54(6):1097.

[6] Iaffaldano M, Muchinsky P. Job satisfaction and job performance. Am Psychol Assoc Inc. 1985;97:251–73.

[7] Judge TA, Thoresen CJ, Bono JE, Patton GK. The job satisfaction-job performance relationship: a qualitative and quantitative review. Psychol Bull. 2001;127(3):376–407. doi: 10.1037/0033-2909.127.3.376 11393302

[8] Redmond P, McGuinness S. Explaining the gender gap in job satisfaction. Applied Economics Letters. 2019;27(17):1415–8. doi: 10.1080/13504851.2019.1686111

[9] Cavanagh TM, Kraiger K, L Henry K. Age-Related Changes on the Effects of Job Characteristics on Job Satisfaction: A Longitudinal Analysis. Int J Aging Hum Dev. 2020;91(1):60–84. doi: 10.1177/0091415019837996 30897924

[10] Morgeson FP, Humphrey SE. Job and team design: Toward a more integrative conceptualization of work design. Research in Personnel and Human Resources Management. 2008:39–91. doi: 10.1016/s0742-7301(08)27002-7

[11] Warr P. Work, Happiness, and Unhappiness. New York, NY: Routledge; 2007.

[12] Riketta M. The causal relation between job attitudes and performance: a meta-analysis of panel studies. J Appl Psychol. 2008;93(2):472–81. doi: 10.1037/0021-9010.93.2.472 18361647

[13] Peiró JM, Montesa D, Soriano A, Kozusznik MW, Villajos E, Magdaleno J, et al. Revisiting the Happy-Productive Worker Thesis from a Eudaimonic Perspective: A Systematic Review. Sustainability. 2021;13(6):3174. doi: 10.3390/su13063174

[14] Peiró JM, Ayala Y, Tordera N, Lorente L. Bienestar Sostenible en el Trabajo: Revisión y Reformulación. Papeles del Psicólogo. 2014;35: 5–14.

[15] Grant P, McGhee P. Hedonic Versus (True) Eudaimonic Well-Being in Organizations BT - The Palgrave Handbook of Workplace Well-Being. In: Dhiman SK, editor. Cham: Springer International Publishing; 2021. pp. 925–943.

[16] Ayala Y, Peiró Silla JMa, Tordera N, Lorente L, Yeves J. Job Satisfaction and Innovative Performance in Young Spanish Employees: Testing New Patterns in the Happy-Productive Worker Thesis—A Discriminant Study. J Happiness Stud. 2016;18(5):1377–401. doi: 10.1007/s10902-016-9778-1

[17] Hackman JR, Oldham GR. The Job Diagnostic Survey: An instrument for the diagnosis of jobs and the evaluation of job redesign projects. New Heaven, Ct.; 1974. Available from: http://oai.dtic.mil/oai/oai?verb=getRecord&metadataPrefix=html&identifier=AD0779828

[18] Oldham GR, Fried Y. Job design research and theory: Past, present and future. Organizational Behavior and Human Decision Processes. 2016;136:20–35. doi: 10.1016/j.obhdp.2016.05.002

[19] Humphrey SE, Nahrgang JD, Morgeson FP. Integrating motivational, social, and contextual work design features: a meta-analytic summary and theoretical extension of the work design literature. J Appl Psychol. 2007;92(5):1332–56. doi: 10.1037/0021-9010.92.5.1332 17845089

[20] Morgeson FP, Garza AS, Campion MA. Work Designs. 2nd ed. Handbook of psychology: Industrial and organizational psychology. 2nd ed. Hoboken, NJ: John Wiley & Sons; 2012. p. 525–559.

[21] Grant AM, Fried Y, Juillerat T. Work matters: Job design in classic and contemporary perspectives. In: Zedeck S, editor. APA handbook of industrial and organizational psychology, Vol 1: Building and developing the organization. Washington, DC, US: American Psychological Association; 2010. p. 417–453.

[22] Morgeson FP, Humphrey SE. The Work Design Questionnaire (WDQ): developing and validating a comprehensive measure for assessing job design and the nature of work. J Appl Psychol. 2006;91(6):1321–39. doi: 10.1037/0021-9010.91.6.1321 17100487

[23] Holman D, Axtell C. Can job redesign interventions influence a broad range of employee outcomes by changing multiple job characteristics? A quasi-experimental study. J Occup Health Psychol. 2016;21(3):284–95. doi: 10.1037/a0039962 26641482

[24] Hackman JR, Oldham GR. Work Redesign. Reading, MA: Addison-Wesley; 1980.

[25] Bayona JA, Caballer A, Peiró JM. The Relationship between Knowledge Characteristics’ Fit and Job Satisfaction and Job Performance: The Mediating Role of Work Engagement. Sustainability. 2020;12(6):2336. doi: 10.3390/su12062336

[26] Warr P. Jobs and Job-holders: Two Sources of Happiness and Unhappiness. In: David S, Boniwell I, Conley Ayers A, editors. The Oxford Handbook of Happiness. Oxford University Press; 2013. pp. 733–750. doi: 10.1093/oxfordhb/9780199557257.013.0054

[27] Besen E, Matz-Costa C, Brown M, Smyer MA, Pitt-Catsouphes M. Job characteristics, core self-evaluations, and job satisfaction: what’s age got to do with it?. Int J Aging Hum Dev. 2013;76(4):269–95. doi: 10.2190/AG.76.4.a 23855183

[28] Hofstede G. Culture’s Consequences: Comparing Values, Behaviors, Institutions and Organizations Across Nations. Thousand Oaks, CA: SAGE Publications, Inc; 2001.

[29] Hackman JR, Oldham GR. Motivation through the design of work: test of a theory. Organizational Behavior and Human Performance. 1976;16(2):250–79. doi: 10.1016/0030-5073(76)90016-7

[30] Dunn WS, Mount MK, Barrick MR, Ones DS. Relative importance of personality and general mental ability in managers’ judgments of applicant qualifications. J Appl Psychol. 1995;80(4):500–9. doi: 10.1037/0021-9010.80.4.500 7642460

[31] Latham GP, Pinder CC. Work motivation theory and research at the dawn of the twenty-first century. Annu Rev Psychol. 2005;56 485–516. doi: 10.1146/annurev.psych.55.090902.142105 15709944

[32] Clark AE. Job satisfaction and gender: Why are women so happy at work?. Labour Economics. 1997;4(4):341–72. doi: 10.1016/s0927-5371(97)00010-9

[33] Donohue SM, Heywood JS. Job satisfaction and gender: an expanded specification from the NLSY. International Journal of Manpower. 2004;25(2):211–38. doi: 10.1108/01437720410536007

[34] Hauret L, Williams DR. Cross‐National Analysis of Gender Differences in Job Satisfaction. Industrial Relations. 2017;56(2):203–35. doi: 10.1111/irel.12171

[35] Fila MJ, Purl J, Griffeth RW. Job demands, control and support: Meta-analyzing moderator effects of gender, nationality, and occupation. Human Resource Management Review. 2017;27(1):39–60. doi: 10.1016/j.hrmr.2016.09.004

[36] Magee W. Effects of Gender and Age on Pride in Work, and Job Satisfaction. J Happiness Stud. 2014;16(5):1091–115. doi: 10.1007/s10902-014-9548-x

[37] Kaiser LC. Gender‐job satisfaction differences across Europe. International Journal of Manpower. 2007;28(1):75–94. doi: 10.1108/01437720710733483

[38] Sloane PJ, Williams H. Job Satisfaction, Comparison Earnings, and Gender. Labour. 2000;14(3):473–502. doi: 10.1111/1467-9914.00142

[39] Bender KA, Donohue SM, Heywood JS. Job satisfaction and gender segregation. Oxford Economic Papers. 2005;57(3):479–96. doi: 10.1093/oep/gpi015

[40] Warr P. Happiness and Mental Health. The Handbook of Stress and Health. Wiley; 2017. p. 57–74. doi: 10.1002/9781118993811.ch4

[41] Warr P. Fuentes de felicidad e infelicidad en el trabajo: una perspectiva combinada. Revista de Psicología del Trabajo y de las Organizaciones. 2013;29(3):99–106. doi: 10.5093/tr2013a15

[42] Bouville G, Dello Russo S, Truxillo D. The moderating role of age in the job characteristics–absenteeism relationship: A matter of occupational context?. J Occupat & Organ Psyc. 2017;91(1):57–83. doi: 10.1111/joop.12188

[43] Kanfer R, Ackerman PL. Aging, Adult Development, and Work Motivation. The Academy of Management Review. 2004;29(3):440. doi: 10.2307/20159053

[44] Ng TWH, Feldman DC. THE RELATIONSHIPS OF AGE WITH JOB ATTITUDES: A META-ANALYSIS. Personnel Psychology. 2010;63(3):677–718. doi: 10.1111/j.1744-6570.2010.01184.x

[45] Carstensen LL, Isaacowitz DM, Charles ST. Taking time seriously. A theory of socioemotional selectivity. Am Psychol. 1999;54(3):165–81. doi: 10.1037//0003-066x.54.3.165 10199217

[46] Zacher H, Frese M. Remaining time and opportunities at work: Relationships between age, work characteristics, and occupational future time perspective. Psychol Aging. 2009;24(2):487–93. doi: 10.1037/a0015425 19485664

[47] Kooij DTAM, De Lange AH, Jansen PGW, Kanfer R, Dikkers JSE. Age and work‐related motives: Results of a meta‐analysis. J Organ Behavior. 2011;32(2):197–225. doi: 10.1002/job.665

[48] United Nations. International Standard Industrial Classification of All Economic Activities (ISIC), Rev.4. New York; 2008. Available from: http://unstats.un.org/unsd/publication/seriesM/seriesm_4rev4e.pdf

[49] Peiró JM, Bayona JA, Caballer A, Di Fabio A. Importance of work characteristics affects job performance: The mediating role of individual dispositions on the work design-performance relationships. Personality and Individual Differences. 2020;157 109808. doi: 10.1016/j.paid.2019.109808

[50] Hair JF, William C, Babin BJ, Anderson RE. Multivariate Data Analysis. 7th ed. Harlow, Essex: Pearson; 2014.

[51] Bayona JA, Caballer A, Peiró J-M. The Work Design Questionnaire: Spanish version and validation. Revista de Psicología del Trabajo y de las Organizaciones. 2015;31(3):187–200. doi: 10.1016/j.rpto.2015.06.001

[52] Van Laar S, Braeken J. Understanding the comparative fit index: It’s all about the base!. Practical Assessment, Research & Evaluation. 2021;26:1–23. doi: 10.7275/23663996

[53] Campion MA. Interdisciplinary approaches to job design: A constructive replication with extensions. Journal of Applied Psychology. 1988;73(3):467–81. doi: 10.1037/0021-9010.73.3.467

[54] Liden RC, Wayne SJ, Stilwell D. A longitudinal study on the early development of leader-member exchanges. Journal of Applied Psychology. 1993;78(4):662–74. doi: 10.1037/0021-9010.78.4.662

[55] DeNisi AS, Murphy KR. Performance appraisal and performance management: 100 years of progress?. J Appl Psychol. 2017;102(3):421–33. doi: 10.1037/apl0000085 28125265

[56] Brislin RW. Translation and content analysis of oral and written material. In: Triandis HC, Berry JW, editors. Handbook of Cross-cultural Psychology, Vol 2. Boston: Allyn and Bacon; 1980. p. 389–444.

[57] Bartels AL, Peterson SJ, Reina CS. Understanding well-being at work: Development and validation of the eudaimonic workplace well-being scale. PLoS One. 2019;14(4):e0215957. doi: 10.1371/journal.pone.0215957 31022285 PMC6483236

[58] Costanza DP, Badger JM, Fraser RL, Severt JB, Gade PA. Generational Differences in Work-Related Attitudes: A Meta-analysis. J Bus Psychol. 2012;27(4):375–94. doi: 10.1007/s10869-012-9259-4

[59] Kent P, Jensen RK, Kongsted A. A comparison of three clustering methods for finding subgroups in MRI, SMS or clinical data: SPSS TwoStep Cluster analysis, Latent Gold and SNOB. BMC Med Res Methodol. 2014;14:113. doi: 10.1186/1471-2288-14-113 25272975 PMC4192340

[60] Podsakoff PM, MacKenzie SB, Lee J-Y, Podsakoff NP. Common method biases in behavioral research: a critical review of the literature and recommended remedies. J Appl Psychol. 2003;88(5):879–903. doi: 10.1037/0021-9010.88.5.879 14516251

[61] Warr P. Work, Happiness, ana Unhappiness. New York: Taylor & Francis; 2007.

