# Supplementary material for: Happy-Productive worker thesis: The role of work characteristics, gender, and age
Source: PLoS One. 2025 Mar 6;20(3):e0316656. doi: 10.1371/journal.pone.0316656 (PMC11884672; doi:10.1371/journal.pone.0316656)
Supplement: S1 Table — (DOCX) [file pone.0316656.s002.docx]

S1 Table. Standardized Regression Weights for the WDQ

| Auto9 | <--- | Autonomy | .674* |
| --- | --- | --- | --- |
| Auto8 | <--- | Autonomy | .826* |
| Auto7 | <--- | Autonomy | .601* |
| Auto6 | <--- | Autonomy | .548* |
| Auto5 | <--- | Autonomy | .706* |
| Auto4 | <--- | Autonomy | .707* |
| Auto3 | <--- | Autonomy | .529* |
| Auto2 | <--- | Autonomy | .751* |
| Auto1 | <--- | Autonomy | .766* |
| TaskVar1 | <--- | Task Variety | .722* |
| TaskVar2 | <--- | Task Variety | .595* |
| TaskVar3 | <--- | Task Variety | .723* |
| TaskVar4 | <--- | Task Variety | .674* |
| TaskSig1 | <--- | Task Significance | .467* |
| TaskSig2 | <--- | Task Significance | .250* |
| TaskSig3 | <--- | Task Significance | .753* |
| TaskSig4 | <--- | Task Significance | .853* |
| TaskID1 | <--- | Task Identity | .729* |
| TaskID2 | <--- | Task Identity | .678* |
| TaskID3 | <--- | Task Identity | .604* |
| TaskID4 | <--- | Task Identity | .849* |
| SkillVar1 | <--- | Skill Variety | .723* |
| SkillVar2 | <--- | Skill Variety | .712* |
| SkillVar3 | <--- | Skill Variety | .736* |
| SkillVar4 | <--- | Skill Variety | .712* |
| FeedJob1 | <--- | Feedback from Job | .602* |
| FeedJob2 | <--- | Feedback from Job | .676* |
| FeedJob3 | <--- | Feedback from Job | .573* |
| JobComp1 | <--- | Job Complexity | .207* |
| JobComp2 | <--- | Job Complexity | .681* |
| JobComp3 | <--- | Job Complexity | .854* |
| JobComp4 | <--- | Job Complexity | .760* |
| InfoProc1 | <--- | Information Processing | .634* |
| InfoProc2 | <--- | Information Processing | .651* |
| InfoProc3 | <--- | Information Processing | .386* |
| InfoProc4 | <--- | Information Processing | .637* |
| ProbSol4 | <--- | Problem Solving | .474* |
| ProbSol3 | <--- | Problem Solving | .474* |
| ProbSol2 | <--- | Problem Solving | .489* |
| ProbSol1 | <--- | Problem Solving | .375* |
| Special1 | <--- | Specialization | .695* |
| Special2 | <--- | Specialization | .414* |
| Special3 | <--- | Specialization | .751* |
| Special4 | <--- | Specialization | .712* |
| SocSupp1 | <--- | Social Support | .644* |
| SocSupp2 | <--- | Social Support | .766* |
| SocSupp3 | <--- | Social Support | .534* |
| SocSupp4 | <--- | Social Support | .441* |
| SocSupp5 | <--- | Social Support | .629* |
| SocSupp6 | <--- | Social Support | .411* |
| Interdp6 | <--- | Interdependence | .419* |
| Interdp5 | <--- | Interdependence | .428* |
| Interdp4 | <--- | Interdependence | .584* |
| Interdp3 | <--- | Interdependence | .698* |
| Interdp2 | <--- | Interdependence | .594* |
| Interdp1 | <--- | Interdependence | .591* |
| InteracOrg1 | <--- | Interaction outside Organization | .764* |
| InteracOrg2 | <--- | Interaction outside Organization | .602* |
| InteracOrg3 | <--- | Interaction outside Organization | .784* |
| InteracOrg4 | <--- | Interaction outside Organization | .916* |
| FeedOthers1 | <--- | Feedback from others | .635* |
| FeedOthers2 | <--- | Feedback from others | .798* |
| FeedOthers3 | <--- | Feedback from others | .767* |
| Ergo1 | <--- | Ergonomics | .733* |
| Ergo2 | <--- | Ergonomics | .774* |
| Ergo3 | <--- | Ergonomics | .001 |
| PhysDem1 | <--- | Physical Demands | .921* |
| PhysDem2 | <--- | Physical Demands | .847* |
| PhysDem3 | <--- | Physical Demands | .898* |
| WrkCond1 | <--- | Work Conditions | .591* |
| WrkCond2 | <--- | Work Conditions | .592* |
| WrkCond3 | <--- | Work Conditions | .310* |
| WrkCond4 | <--- | Work Conditions | .489* |
| WrkCond5 | <--- | Work Conditions | .553* |
| EquipUse1 | <--- | Equipment Use | .682* |
| EquipUse2 | <--- | Equipment Use | .516* |
| EquipUse3 | <--- | Equipment Use | .657* |

Note. *p < 0.001

S1 Table. Factor loadings for the WDQ (continuation)
