# Supplementary material for: Happy-Productive worker thesis: The role of work characteristics, gender, and age
Source: PLoS One. 2025 Mar 6;20(3):e0316656. doi: 10.1371/journal.pone.0316656 (PMC11884672; doi:10.1371/journal.pone.0316656)
Supplement: S2 Table — (DOCX) [file pone.0316656.s003.docx]

**S2 Table. Factor correlations for the WDQ**

|  | 1 | 2 | 3 | 4 | 5 | 6 | 7 | 8 | 9 | 10 | 11 | 12 | 13 | 14 | 15 | 16 | 17 |
| --- | --- | --- | --- | --- | --- | --- | --- | --- | --- | --- | --- | --- | --- | --- | --- | --- | --- |
| 1. Autonomy | -- |  |  |  |  |  |  |  |  |  |  |  |  |  |  |  |  |
| 1. Task variety | .36^**^ | -- |  |  |  |  |  |  |  |  |  |  |  |  |  |  |  |
| 1. Task significance | .20^**^ | .36^**^ | -- |  |  |  |  |  |  |  |  |  |  |  |  |  |  |
| 1. Task identity | .50^**^ | .15^**^ |  | -- |  |  |  |  |  |  |  |  |  |  |  |  |  |
| 1. Feedback from job | .56^**^ | .39^**^ | .65^**^ |  | -- |  |  |  |  |  |  |  |  |  |  |  |  |
| 1. Job complexity | -.22^**^ | .09^*^ | .10^**^ | -.25^**^ | -.17^**^ | -- |  |  |  |  |  |  |  |  |  |  |  |
| 1. Information processing | .39^**^ | .89^**^ | .51^**^ | .18^**^ | .39^**^ | .36^**^ | -- |  |  |  |  |  |  |  |  |  |  |
| 1. Problem solving | .53^**^ | .72^**^ | .52^**^ | .31^**^ | .52^**^ | .14^**^ | .98^**^ | -- |  |  |  |  |  |  |  |  |  |
| 1. Skill variety | .37^**^ | .71^**^ | .36^**^ | .21^**^ | .29^**^ | .20^**^ | .87^**^ | .82^**^ | -- |  |  |  |  |  |  |  |  |
| 1. Specialization | .37^**^ | .61^**^ | .52^**^ | .30^**^ | .43^**^ | .22^**^ | .93^**^ | .88^**^ | .84^**^ | -- |  |  |  |  |  |  |  |
| 1. Social support | .51^**^ | .24^**^ | .36^**^ | .44^**^ | .55^**^ | -.14^**^ | .41^**^ | .55^**^ | .29^**^ | .32^**^ | -- |  |  |  |  |  |  |
| 1. Interdependence | .10^**^ | .43^**^ | .29^**^ | .18^**^ | .36^**^ | .12^**^ | .54^**^ | .52^**^ | .37^**^ | .45^**^ | .17^**^ | -- |  |  |  |  |  |
| 1. Interaction outside org. | .20^**^ | .21^**^ | .37^**^ | .10^**^ | .15^**^ | -.09^*^ | .34^**^ | .35^**^ | .18^**^ | .20^**^ | .45^**^ | .12^**^ | -- |  |  |  |  |
| 1. Feedback from others | .23^**^ | .21^**^ | .26^**^ | .37^**^ | .73^**^ | -.12^**^ | .21^**^ | .32^**^ | .16^**^ | .24^**^ | .41^**^ | .21^**^ | .09^*^ | -- |  |  |  |
| 1. Ergonomics | .36^**^ | .17^**^ | .22^**^ | .38^**^ | .41^**^ | -.07^*^ | .31^**^ | .30^**^ | .22^**^ | .22^**^ | .42^**^ | .15^**^ | .05^*^ | .30^**^ | -- |  |  |
| 1. Physical demands | -.03 | .13^**^ | -.06 | .05^*^ | -.02 | -.14^**^ | -.09^*^ | .19^**^ | .13^**^ | .07^*^ | -.02 | .12^**^ | .13^**^ | .01 | -.21^**^ | -- |  |
| 1. Work conditions | .36^**^ | .07^*^ | .24^**^ | .37^**^ | .42^**^ | -.12^**^ | .25^**^ | .26^**^ | .12^**^ | .18^**^ | .44^**^ | .07^*^ | .03 | .28^**^ | .78^**^ | -.31^**^ | -- |
| 1. Equipment use | .09^**^ | .43^**^ | .17^**^ | .20^**^ | .23^**^ | .06^*^ | .46^**^ | .53^**^ | .54^**^ | .54^**^ | .07^*^ | .42^**^ | .14^**^ | .18^**^ | .05 | .52^**^ | -.10^*^ |
